# CAR‐T cells derived from multiple myeloma patients at diagnosis have improved cytotoxic functions compared to those produced at relapse or following daratumumab treatment

**DOI:** 10.1002/jha2.479

**Published:** 2022-06-21

**Authors:** Audrey Abecassis, Nathalie Roders, Maxime Fayon, Caroline Choisy, Elisabeth Nelson, Stephanie Harel, Bruno Royer, Camille Villesuzanne, Alexis Talbot, David Garrick, Michele Goodhardt, Jean‐Paul Fermand, Mike Burbridge, Bertrand Arnulf, Jean‐Christophe Bories

**Affiliations:** ^1^ Université Paris Cité, INSERM, HIPI Paris France; ^2^ Immuno‐Hematology Saint‐Louis Hospital Paris France; ^3^ Oncology Translational and Clinical Research Institut de Recherches Internationales Servier (IRIS) Suresnes France

**Keywords:** CAR‐T cells, daratumumab, multiple myeloma, peripheral tcells, relapse

## Abstract

Chimeric antigen receptor T cells (CAR‐T) have provided promising results in multiple myeloma (MM). However, many patients still relapse, pointing toward the need of improving this therapy. Here, we analyzed peripheral blood T cells from MM patients at different stages of the disease and investigated their phenotype and capacity to generate functional CAR‐T directed against CS1 or B Cell Maturation antigen. We found a decrease in naive T cells and elevated frequencies of exhaustion markers in T cells from treated MM patients. Interestingly, individuals treated with daratumumab display elevated ratios of central memory T cells. CAR‐T derived from patients at relapse show reduced in vitro expansion and cytotoxic capacities in response to MM cells compared to those produced at diagnosis. Of note, CAR‐T from daratumumab treated patients display intermediate defects. Reduced anti‐myeloma activity of CAR T cells from treated patients was also observed in a mouse model. Our findings suggest that T cell defects in MM patients, specifically during relapse, have a major impact on their capacity to generate efficient therapeutic CAR‐T. Selecting naive or central memory T cell subsets to generate therapeutic T cells could improve the CAR‐T therapy for MM.

## 1 INTRODUCTION

The development of novel therapeutic modalities associating proteasome inhibitors, immunomodulatory drugs, and monoclonal antibodies, such as daratumumab, has extended the survival of multiple myeloma (MM) patients [1, 2, 3]. However, MM remains incurable, and more efficient therapies are needed for patients with relapsed and/or refractory (R/R) disease. Chimeric antigen receptor (CAR) T, targeting SLAMF7 (CS1) or the B cell maturation antigen (BCMA) can efficiently eliminate MM cells in vitro [4, 5, 6]. Furthermore, anti‐BCMA CAR‐T immunotherapy has provided good response rates in pretreated/refractory MM patients, however, the median progression free survival remains relatively short, ranging from 11 to 15 months depending on the study [7, 8].

Progression of MM is associated with major changes in the T cell compartment in blood and bone marrow of patients [9]. Decreased CD4/CD8 T cell ratio, elevation of regulatory T cells (Treg) over Th17 T cells, and expression of exhaustion markers have been reported [10]. Furthermore, many MM therapies, such as autologous stem cell transplantation or exposure to Immunomodulatory imide drugs (IMIDs), are thought to impact on the function of T lymphocytes [11]. In line with this, it has recently been suggested that CAR‐T cells manufactured from MM patients following induction therapy could be more efficient than those generated from relapsed individuals [12]. Here, we further investigated the impact of treatments and disease progression on blood T cell populations and on the activity of CAR‐T generated from MM patients at different stage of the disease.

## 2 MATERIALS AND METHODS

Details of patients included in the study are shown in Table S1. Detailed experimental description of sample collection, fluorescence‐activated cell sorting (FACS), production of CAR T cells, and functional assays are provided in Supplementary Information.

## 3 RESULTS AND DISCUSSION

**FIGURE 1:**
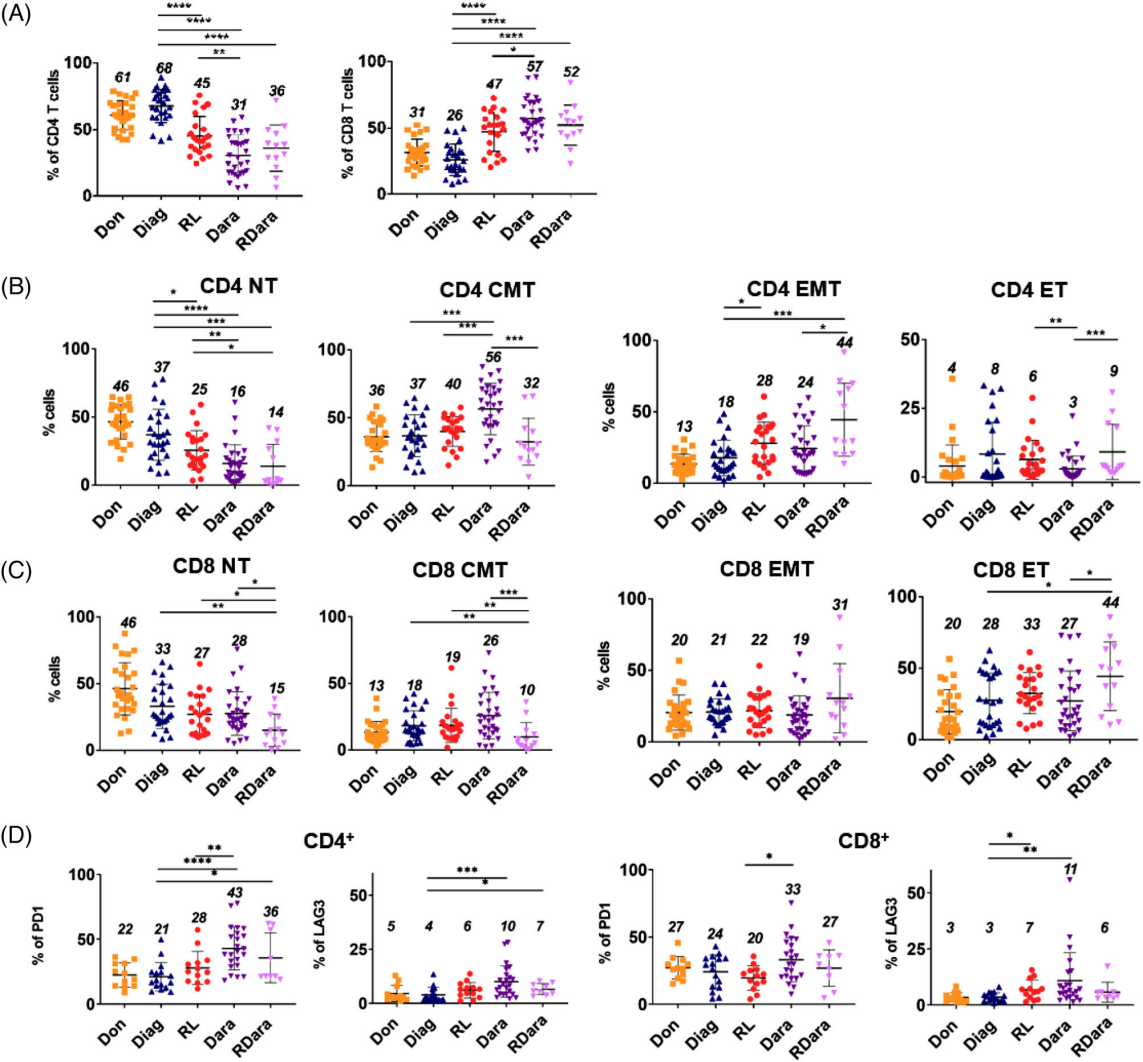
Analysis of T‐cell subsets in the peripheral blood of multiple myeloma (MM) patients. (A) Scatter dot plots show the percentages of CD4 and CD8 expressing T cells in peripheral blood mononuclear cells (PBMCs) isolated from healthy donors (Don, *N* = 27), MM patients at diagnosis (Diag, *N* = 26), at relapse MM (RL, *N* = 23), treated with daratumumab (Dara, *N* = 30), and in relapse after daratumumab treatment (RDara, *N* = 13). (B and C) Scatter dot plots show the percentages of naïve (CD3+, CD62L+, CD45RO‐) (N), effector (CD3+, CD62L‐, CD45RO‐) (E), central memory (CD3+, CD62L+, CD45RO+) (CM) and effector memory (CD3+, CD62L‐, CD45RO+) (EM) among CD4 (B) and CD8 (C) expressing T cells from healthy donors and MM patients as in (A). (D) Scatter dot plots show the percentages of PD1 and LAG3 expressing cells among CD4 or CD8 positive CD3^+^ cells in Don (*N* = 12), Diag (*N* = 16), RL (*N* = 13), Dara (*N* = 23), and RDara (*N* = 9). Numbers in the scatter dot plots indicate the mean percentages of each cohort. *p*‐values between two groups were examined with a Mann‐Whitney *U*‐test. *p ≤ 0.05, **p ≤ 0.005, ***p ≤ 0.001

To directly investigate T cell populations in MM, we prospectively constituted four cohorts of patients at diagnosis (Diag), at relapse (RL), upon daratumumab treatment (Dara) and at relapse after daratumumab treatment (RDara) (patient characteristics are detailed in Table S1A), as well as from non age‐matched healthy donors (Don). As previously reported [11], FACS analysis on peripheral blood mononuclear cells from RL patients showed that the ratios of CD4/CD8 T cell were inverted compared to healthy donors and Diag patients. However, we observed that this defect was amplified in the Dara and RDara cohorts (Figure 1A) suggesting that antibody treatments worsen this feature. Analysis of CD45RO and CD62L expression revealed that the percentages of CD4^+^CD62L^+^CD45RO^–^ naïve T cells (NT) in RL were reduced compared to Diag (25% vs 37%), and this reduction was more pronounced in Dara and RDara patients (16% and 14% respectively) (Figure 1B). Conversely, the percentages of central memory (CM) CD4^+^CD62L^+^CD45RO^+^ cells were significantly higher in Dara patients (56%) than in the other groups (32%–40%), while the frequency of effector memory (EM) CD4^+^CD62L^–^CD45RO^+^ cells was elevated in RL (28%) and RDara (44%) compared to Diag samples (18%). Peripheral CD8^+^ T cell populations tended to display similar defects, with decreased percentages of NT in all cohorts of treated patients (28%–15%) compared with Diag (33%), elevated frequencies of CM in Dara (26% vs 18%), and of effector (ET) in RDara patients (44% vs 28%) (Figure 1C). Furthermore, analysis of PD‐1 and LAG3, whose expression correlates with loss of T cell cytotoxicity showed that CD4^+^ T cells from Dara and RDara patients expressed higher percentages of both exhaustion markers compared to Diag (43% and 36% vs 21% for PD1 and 10% and 7% vs 4% for LAG3) (Figure 1D). Altogether, our results indicate that reduced naïve populations and elevated frequencies of exhaustion markers are common features of T cells in treated MM patients. However, daratumumab‐treated patients display specific alterations in T‐cell subsets, such as elevated ratio of CM T cells, while those who relapse after daratumumab therapy have increased frequencies of effector subsets.

**FIGURE 2:**
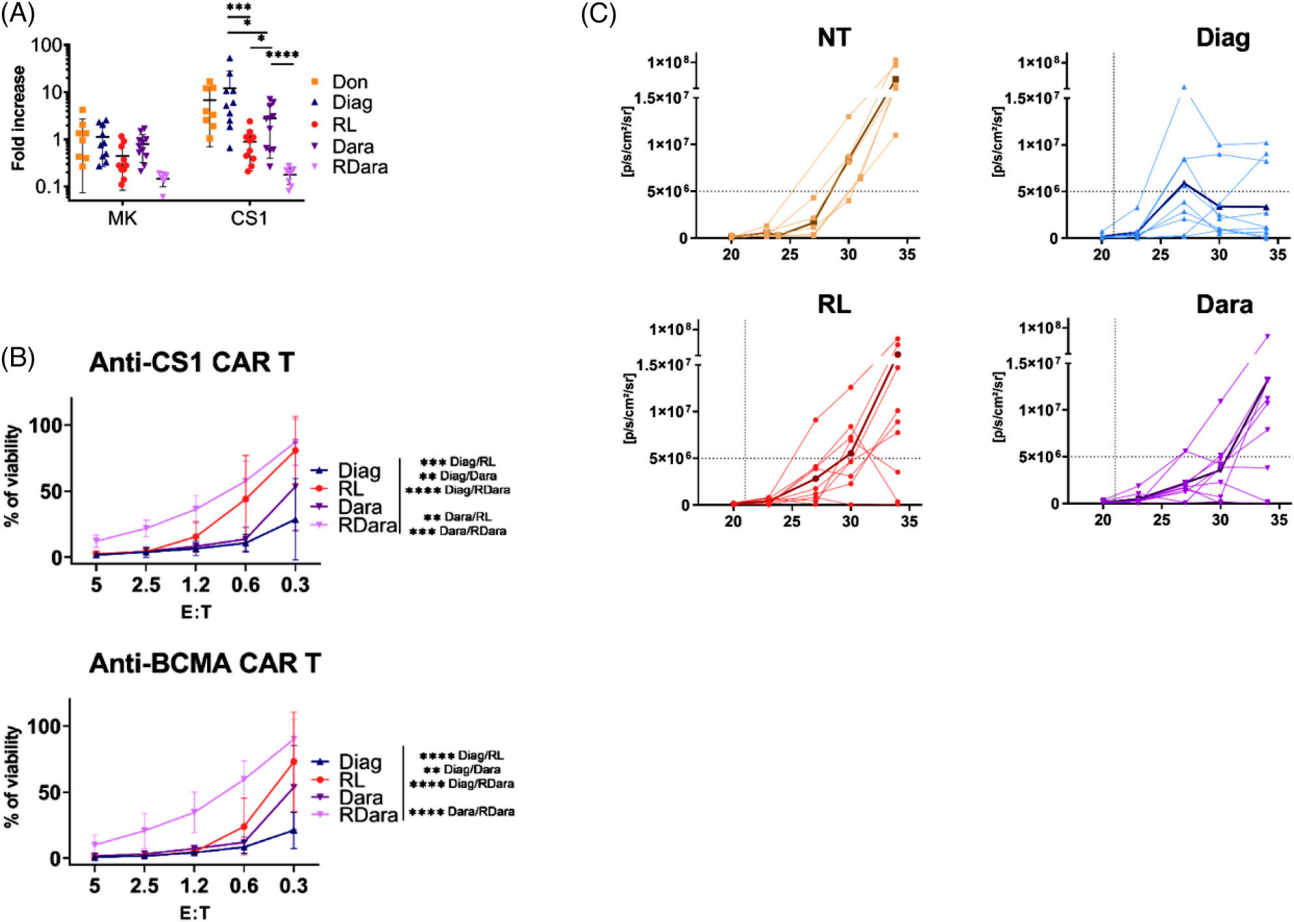
Functional analysis of chimeric antigen receptor T cells (CAR‐T) from multiple myeloma (MM) patients. (A) Scatter dot plot showing expansion (total number/starting number) of CAR‐T cells cultured for 7 days on NIH3T3 cells expressing the CS1 antigen. Anti‐CS1 CAR‐T (CS1) or nonfunctional CAR‐T (MK) generated from donors (Don, N = 8) and MM patients at diagnosis (Diag, *N* = 10), in relapse (RL, *N* = 10), treated with dartumumab (Dara, *N* = 11) or in relapse after daratumumab treatment (RDara, *N* = 8) were expanded with anti‐CD3/anti‐CD28 beads and interleukine‐2 (IL‐2) for 6 days, then separated from beads/IL‐2, and 5.10^4^ cells were plated on feeders. (B) In vitro anti‐MM activity of anti‐CS1 and anti‐BCMA CAR‐T cells from MM patients. Curves represent the percentage of viable MM1.S cells in the culture compared to controls with no specific killing. 2.5 × 10^4^ luciferase expressing MM1.S cells (Target:T) were co‐cultured with anti‐CS1 CAR‐T (Effector: E) from MM patients, at various E:T ratios for 24 h. Anti‐CS1 CAR‐T were generated from patients at diagnosis (Diag, *N* = 8), at relapse (RL, *N* = 8), upon daratumumab (Dara, *N* = 9) or in relapse after daratumumab treatment (RDara, *N* = 8). Anti‐B cell maturation antigen (BCMA) CAR‐T were generated from patients at diagnosis (Diag, N = 4), at relapse (RL, N = 4), upon daratumumab (Dara, N = 6) or in relapse after daratumumab treatment (RDara, N = 8). *p*‐values were examined with a two‐way ANOVA test (*p < 0.05; **p < 0.01; ***p < 0.001; ****p < 0.0005). (C) In vivo activity of anti‐CS1 CAR‐T generated from MM patients in the MM1.Sluc Xenograft mouse model. Shown are the longitudinal radiance levels (luciferase activity) measured in mice injected with CAR‐T (dotted line) from MM patients (Diag, RL, Dara) or left untreated (NT). Six to 12‐week‐old NOD/SCID/IL‐2Rγnull mice were inoculated with 5 × 10^6^ MM1.SLuc cells by tail vein injection at day 0, followed, 21 days later, by infusion of 10^6^ CAR‐T. Bioluminescence was measured with the IVIS Imaging System at day 20, 23, 26, 30, and 34 after tumor injection. Bold curves represent the average radiances for each cohort. Radiance was measured on the entire body of mice. These experiments were performed with CAR‐T generated from three independent patients of each group, each injected into three independent mice (nine mice per group)

Given these phenotypic alterations, we investigated the function of anti‐CS1 CAR‐T^5^ generated by lentiviral transduction of peripheral T cells from MM patients included in each cohort (see Table S1B for patient characteristics). We first established an in vitro assay to measure the expansion of the anti‐CS1 or scFv deficient (Mock) CAR‐T cells cultured on CS1 expressing NIH3T3 cells. After 7 days in culture, we observed no proliferation of CAR‐T derived from RL, while those generated from Diag and Don expanded 12 and 8 fold, respectively (Figure 2A). Interestingly, CAR‐T produced from Dara patients expanded around three‐fold, whereas those derived from RDara cohort displayed no sign of proliferation. We next analyzed the potential of anti‐CS1 CAR‐T, produced from MM patients (see Table S1C for patient characteristics), to kill luciferase expressing MM1.S MM cell line (MM1.Sluc) in vitro. Luciferase levels, which reflect the number of live MM cells, were measured after overnight co‐cultures of anti‐CS1 CAR‐T (effector: E) and MM1.Sluc (target: T) mixed at ratios varying from 5:1 to 1:3. To calculate the viability of MM cells, luciferase levels measured at the different E:T ratios were normalized to the luminescence values obtained in co‐cultures with control CAR‐T cells that do not induce specific killing. At high E:T ratios, all anti‐CS1 CAR‐T readily killed MM1.S cells (Figure 2B). However, at the lower E:T ratio of 1:3, the capacity to kill MM cells was lower for CAR‐T generated from RL and RDara patients (81% and 87% cell viability, respectively) compared to those produced from Diag (29% cell viability). Of note, CAR‐T generated from the Dara group showed intermediate killing levels (53% cell viability). To test if this defect was also observed for other targets, we constructed an anti‐BCMA CAR and tested the cytotoxic activity of T cells from all four groups of MM patients transduced with this construct on MM1.S cells expressing luciferase, as before. In line with our previous results, we found that at a 1:3 E:T ratio, anti‐BCMA CAR‐T from RL and RDara displayed weaker cytotoxic activity against MM cells (cell viability 73% and 90%, respectively) compared to Diag (21%) and Dara patients (54%) (Figure 2B). In order to investigate the activity of CAR‐T from MM patients in vivo, we developed a xenograft mouse model by inoculating MM1.Sluc cells into immune‐deficient mice and followed luciferase levels with an IVIS Imaging System [13]. Twenty‐one days after MM1.S injection, anti‐CS1 CAR‐T from Diag, RL, or Dara patients (characteristics in Table S1C) were transplanted intravenously into engrafted mice and bioluminescence measured every 3 or 4 days. Tumor progressed rapidly in mice that received no therapeutic T cells with an average luminescence of 44.7 × 10^6^ *p/s/cm^2^/sr* at day 34 posttransplantation (Figure 2C). Remarkably, injection of CAR‐T from Diag readily controlled tumor growth over time with an average luminescence below 3.3 × 10^6^ *p/s/cm^2^/sr* at day 34. In contrast, treatment with CAR‐T from Dara or RL patients showed intermediate MM growth characterized by average luminescence levels of 13 × 10^6^ *p/s/cm^2^/sr* and 18 × 10^6^ *p/s/cm^2^/sr*, respectively at this time point. Thus, relative to CAR‐T cells derived from patients at diagnosis, those generated from treated patients display weaker killing activity in vitro and are less effective in controlling tumor progression in vivo.

Altogether our results reveal profound defects in the peripheral T cell populations in relapse MM patients as well as in those treated with daratumumab. We show that CAR‐T generated from relapse MM patients exhibit reduced proliferative and killing activities compared to those derived from patients at diagnosis. These findings could suggest that the decrease in naive T cells observed in all groups of treated patients may impact the production of efficient autologous CAR‐T in MM. Moreover, we show that CAR‐T cells from daratumumab treated patients are more efficient in vitro than those generated from patients at relapse. Since Dara patients have all experienced a first relapse prior to daratumumab therapy, this observation suggests that reduced anti‐myeloma activity of CAR‐T from relapse patients may be reversible. Altogether, our results indicate that manufacturing CAR‐T during remission phases or from naïve or central memory cells could help to improve CAR‐T therapy in Myeloma.

## CONFLICT OF INTEREST

The authors declare no competing financial interests and have no conflict of interest to disclose.

## AUTHOR CONTRIBUTIONS

J C B, M B, and B A designed the study. A A, N R, M F, C C, and E N performed experiments. S H, B R, C V, A T, J P F, and B A provided samples and clinical data from patients. A A, N R, M G, D G, B A, and J C B analyzed the data. J C B wrote the manuscript. All authors read and approved the final manuscript.

## ETHICS STATEMENT

Peripheral blood samples from healthy donors were obtained from the Etablissement Francais du Sang (EFS). Peripheral blood samples from MM patients were collected in the department of Immuno‐Hematology of the Saint‐Louis Hospital using standard procedures, as part of routine diagnostic work‐up. Written informed consent for research was provided by the patients in accordance with the Declaration of Helsinki and French law. The protocol received approval of the ethical committee “GHU Nord” number:23798‐2020012709469025 121.

## Supporting information

Supplementary Table: Demographics and clinical characteristics of MM patients included in phenotypic analysis (A), proliferation (B), in vitro (C) and in vivo (D) killing assays
