# Supplementary material for: CAR‐T cells derived from multiple myeloma patients at diagnosis have improved cytotoxic functions compared to those produced at relapse or following daratumumab treatment
Source: EJHaem. 2022 Jun 21;3(3):970–4. doi: 10.1002/jha2.479 (PMC9421998; doi:10.1002/jha2.479)
Supplement: Supplementary file 1 — Supplementary Table: Demographics and clinical characteristics of MM patients included in phenotypic analysis (A), proliferation (B), in vitro (C) and in vivo (D) killing assays [file JHA2-3-970-s001.docx]

**Supplementary information**

**Supplementary Table**


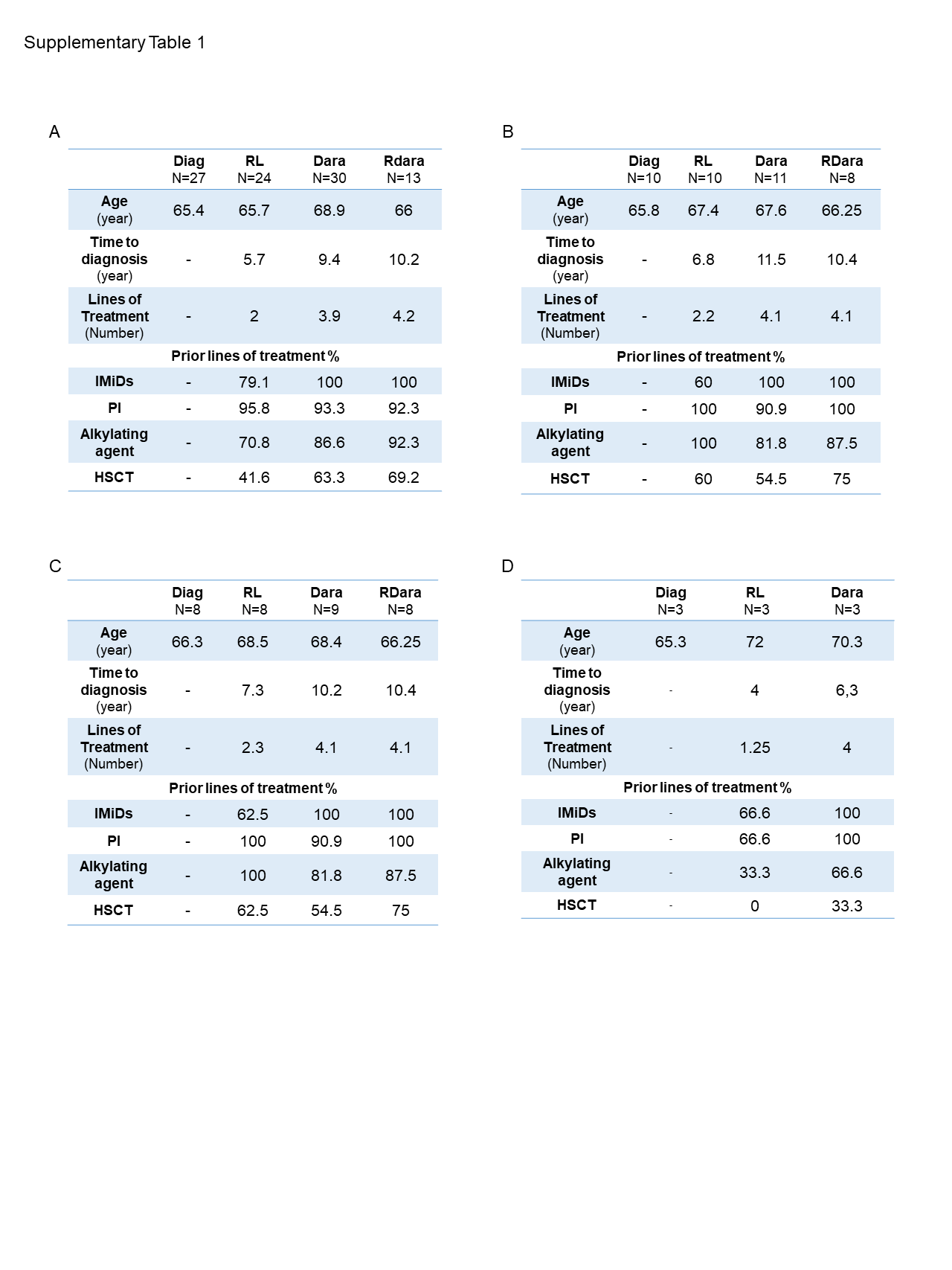


Supplementary Table: Demographics and clinical characteristics of Multiple Myeloma patients included in phenotypic analysis (A), proliferation (B), *in vitro* (C) *and in vivo* (D) killing assays. **In (A), the median age of patients was 66.5 years (range, 40 to 86), and the median time since diagnosis was 5.7 years (range, 1 to 15) for RL, 9.2 years (range, 5 to 23) for Dara and 10.2 years (range, 4 to 16) for RDara patients. The median number of previous regimens was 2 (range, 1 to 3, with 20 patients receiving 1 or 2 lines) among RL patients, 3.9 (range, 3 to 6) among Dara and 4.2 (range, 3 to 7) among RDara. The great majority of treated patients had previously received both a proteasome inhibitor and an immunomodulatory agent.**

**Materials and Methods:**

**Patients and blood samples.** Peripheral blood samples from healthy donors and myeloma patients were obtained from the Etablissement Francais du Sang and the department of Immuno-Hematology (Hopital Saint-Louis, Paris), respectively. In all cases, informed consent of patients and volunteers were obtained in accordance with the Declaration of Helsinki and with approval of the Saint-Louis Hospital Internal Review Board. **Blood cells from patients at daratumumab were collected at least 4 months after the last line of treatment (daratumumab, bortezomib, thalidomide, and dexamethasone) and at least 3 weeks after the last injection of the therapeutic antibody.** T cells were isolated from blood samples with the Pan T cell isolation Kit Human (Milteniy Biotec), activated in RPMI-1640 supplemented with 10% fetal bovine serum, Penicillin/Streptomycin, Glutamine 2mM (all from Invitrogen), IL2 (5ng/mL) and beads anti-CD3/CD28 Dynabeads® (Thermofisher).

**Flow Cytometry analyses.** Peripheral Blood Mononuclear Cells (PBMC) were isolated by Pancoll®, stained with Fixable viability dye, anti-CD3, anti-CD4, anti-CD8, anti-CD62L, anti-CD45RO antibodies (Biolegend). All FACS acquisition was performed on a LSR Fortessa (Beckon Dickinson) and data were analyzed with Flowjo 10.

**Vector constructs and lentivirus production.** The anti-CS1 CAR has been previously published^15^. The anti-BCMA CAR was constructed as follows: an anti-BCMA specific scFv fragment was generated from the clone CA8 (https://patents.justia.com/patent/20140105915) and fused to a human CD8 hinge/transmembrane region and 4-1BB co-stimulatory and a CD3 ζ-chain signaling domains. The mock control CAR construct contained CD28 and 4-1BB co-stimulatory linked to the CD3 ζ-chain signaling domains, but lacked the scFv domain. CAR cDNAs were cloned in a modified pRRL lentiviral vector (Addgen #12252) allowing transgene expression from the sFFV promoter and GFP expression from the PGK promoter. Lentiviral stocks were prepared by transient co-transfection in human 293T cells of three plasmids (the CAR vector plasmid, the packaging plasmid psPAX2, and the VSV-G protein envelope plasmid pMD2.G). Seventy-two hours after transfection, viral stocks were concentrated by ultracentrifugation and stored at –80°C.

**CAR-T cell production.** T cells were purified from buffy coat of blood samples using anti-CD3 coated beads (Miltenyi) and activated with anti-CD3/CD28 beads (Thermofisher) in CTS OpTmizer medium (Thermofisher) the presence of IL-2 (10µg/mL). Twenty-four hours after activation, T cells were transduced with lentiviral supernatants on retronectin (Takara)-coated plates. Transduction efficiency was measured by FACS based on GFP expression and ranged between 60 to 85% after 6 days. Transduced T cells were further expanded in IL-2 and anti-CD3/CD28 beads containing culture medium. Then, IL-2 and beads were removed at day 6 for proliferation assay. For cytotoxic assay, beads were removed at day 10 and IL-2 at day 15.

**Luciferase-based cytotoxicity assay.** Bulk cytotoxicity of CAR-T cells and controls was determined by luciferase-based cytotoxic assay. MM1.S cells expressing Fire Fly luciferase were co-cultured in 96-well plates with CAR-T cells at different E:T ratios for 24 h. After 24h of co-culture, luciferase substrate (Bright-Glo, Promega) was added to each well. Emitted light was detected in a luminescence plate reader Clariostar (BMG Labtech).

***In vitro* CAR-T cell expansion assays.** At day 6 after transduction, activation beads and IL-2 were removed and CAR-T cells from the various cohorts were cultured on Mitomycin-C treated feeders (wild type or CS1 expressing NIH3T3) at a ratio 1:2. CAR-T cells were passed on fresh feeder cells every 24h or 48h. After one week, cells were analyzed by flow cytometry (Fortessa, BD) with Brightcount beads (ThermoFisher) to measure the numbers of live cells. Analysis were performed with FlowJo10.

***In vivo* CAR-T cell function**. Six to 12-week-old NOD/SCID/IL-2Rγnull mice were inoculated with 5x10^6^ MM1.SLuc cells by tail vein injection (i.v) at day 0, followed, 21 days later, by infusion of 10^6^ CAR-T. Bioluminescence was measured with the IVIS Imaging System (PerkinElmer) at day 20, 23, 26, 30 and 34 after tumor injection.

**Statistics**. All experimental data are presented as mean ± s.d. Appropriate statistical tests were used to analyze data, as described in the figure legends. Statistical analysis was performed on GraphPad Prism 7 software.
